# Impact of Diabetic Foot Ulcer on the Health-Related Quality of Life of Diabetic Patients in Khartoum State

**DOI:** 10.7759/cureus.52813

**Published:** 2024-01-23

**Authors:** Yusra H Hamid, Mathani Mohammed, Safaa Hamid, Wiaam Mohamedahmed, Osama Ahmed

**Affiliations:** 1 Community Medicine, Faculty of Medicine, University of Khartoum, Khartoum, SDN; 2 General Surgery, Sudan Medical Specialization Board, Khartoum, SDN; 3 Community Medicine and Public Health, National University, Khartoum, SDN

**Keywords:** quality of life, sudan khartoum, dfu (diabetic foot ulcer), health-related quality of life (hrqol), dsf

## Abstract

Background

This is a novel study from Sudan aimed at comparing health-related quality of life (HRQoL) between diabetic foot ulcer (DFU) patients and diabetes patients without DFU. Additionally, this study aimed to determine the factors correlating with lower HRQoL.

Methodology

A descriptive, cross-sectional study with a comparative group was conducted in three diabetes centers in Khartoum, Sudan, in 2020. A total of 120 Sudanese diabetic patients (mean age = 52 years) were divided into two groups, without DFU and with DFU, and interviewed in person. Demographic and clinical variables were recorded. HRQoL was evaluated using the standardized RAND-36 (36-Item Short Form Health Survey) survey for all participants. HRQoL domains and total scores were compared in the two groups using the t-test. Inference against sociodemographic data was determined using Pearson’s test and analysis of variance.

Results

The DFU group (36 males, 24 females) scored significantly lower in five (yet higher in two out of the eight subscales) compared to the non-DFU diabetic group (31 males, 29 females). Energy/fatigue levels remained insignificant. Being a female (p = 0.03), painful ulcers (p = 0.001), insulin use (p = 0.04), and newly developed ulcers (p = 0.005) were associated with lower HRQoL total scores in the DFU group. However, educational levels had a positive correlation (p = 0.02).

Conclusions

DFU patients have lower HRQoL than diabetic patients without ulcers. They need more support, including disease-specific education, realistic expectations (regarding ulcer’s impact, healing, and management), physical rehabilitation, and culturally sensitive assessment tools.

## Introduction

Diabetes mellitus is a growing public health concern that is affecting people globally and poses a major socioeconomic challenge [1]. Diabetes, when not properly managed, can lead to complications affecting various parts of the body. These include the cardiovascular system, kidneys, and eyes. Chronic ulcers related to diabetes can result in limb amputations. These widespread impacts of diabetes present significant challenges to a patient’s quality of life (QoL) and may restrain the daily activity of patients [2,3].

A diabetic foot, according to the World Health Organization in 2002, is an infection, ulcer, or tissue damage in the lower limb that is accompanied by nerve problems and insufficient blood flow. The diabetic foot ulcer (DFU), a serious consequence of diabetes mellitus, is a typical example of this. About 15% of diabetics have an open wound on their foot, and some of them need hospital care because of an infection or other complications. Hospitalizations for diabetic foot problems are more common in nations with high diabetes prevalence, such as India, South Africa, Brazil, and Tanzania. For diabetics in sub-Saharan Africa, foot ulcers are a serious health risk that can result in high medical expenses and even fatalities [4]. A study from Saudi Arabia found that neuropathy, peripheral arterial disease, disease duration, and underlying osteomyelitis are the major risk factors for developing foot ulcers and need to be addressed while educating patients [5].

Similar to many affluent nations, DFU is a primary disabling complication in poor nations that can result in severe morbidity and even amputations [6,7]. In Sudan, diabetes has emerged as a serious health issue. A 2016 study found a diabetes prevalence of 2.5% in north Sudan’s rural areas compared to an estimated prevalence of 19% in metropolitan areas. Overall, 85% of Sudanese people with type 2 diabetes have uncontrolled diabetes, which is a high prevalence compared to other developed and developing nations. About 18.1% of Sudanese people have diabetic feet, which is higher than anticipated on a regional and global level. For instance, estimates of the prevalence of diabetic foot in Tanzania, Nigeria, and Cameroon, respectively, were 13%, 9.5%, and 15%. Among all risk factors, having diabetes mellitus for more than 10 years (odds ratio (OR) = 1.8, 95% confidence interval (CI) = 1.15-2.8; p = 0.01) and the level of foot care knowledge (OR = 0.2, 95% CI = 0.9-0.4; p < 0.0005) were found to be significant predictors for the development of DFU [8,9].

The mortality rate from diabetic foot infection differs among hospitals in Sudan. Inpatients with diabetic feet have seen a substantial rise in lower limb amputations (LLAs) in Khartoum hospitals in recent years. Presently, 10.2% of all complications reported from private clinics in the Khartoum state are diabetic septic feet. According to some studies, 3.9% of the rural Sudanese population has DFU. The rate of hospital bed occupancy is considerably impacted by this condition. Diabetes and its consequences, such as amputations, have a significant socioeconomic impact. Medication, hospital stays, treatments, and supplies all have direct expenses. Indirect costs borne by patients and their families include lost work time, income loss, diversion of family resources from other basic needs, and premature death. All these factors significantly affect the dependents of the patient [9]. The impact of lower-extremity ulcers on the QoL of patients with diabetes has been assessed in several studies [3,7,10-13], all showing a lowered index when compared to diabetic individuals without foot lesions. An understanding of the specific effects of chronic DFUs on individual patients’ QoL is crucial to the direction of treatment, management of compliance, and patient/practitioner communication. Studies are still needed to determine how significant DFU is in Sudan and to determine whether additional support is needed for this subset of patients.

## Materials and methods

Study design

An observational, descriptive, cross-sectional study with a comparative group was conducted in three diabetes centers across Khartoum which were selected conveniently.

Study population

Patients with diabetes (type I and II) who had foot ulcers at the time of questionnaire administration were divided into two groups. The study group (group 2) included diabetics with DFU, and the comparative group (group 1) included diabetics without DFU.

We included patients who were diagnosed with diabetes (type I or II) based on medical records, were Sudanese residents, and could read or understand Arabic. Those who refused to provide consent, could neither read nor understand Arabic, had non-diabetic foot lesions, and had comorbidities such as renal failure or heart diseases and mental/cognitive problems were excluded.

A random sampling method was applied to enroll participants from each center. The prevalence of DFU (P) was estimated to be 18.1% in Sudan [2]. In total, 120 individuals were recruited for the study, with 60 DFU patients and 60 diabetic patients without DFU as a comparative group.

Sampling technique

A multistage sampling method was used to enroll participants in the study; first, convenient non-probability sampling was used to pick three diabetes centers around Khartoum. Then, random sampling was employed to pick patients for participation from the outpatient or inpatient clinic of each center.

Data collection

General sociodemographic data, including age, gender, occupational and marital status, whether or not they had children, educational level, residence, and social habits such as smoking, were collected from study participants.

Diabetes-related characteristics, including the type (type 1, type 2, or unknown) and duration of diabetes, were obtained from patients and verified through staff interviews or medical record reviews.

Ulcer-related characteristics, including duration, whether ulcers were painful or painless, and a history of previous ulcers or amputation, were also recorded.

Data collection tool

Both the study and comparative groups were interviewed with a structured close-ended, standardized version of the SF-36 General Health Related Quality of Life (GHRQoL) questionnaire known as RAND 36-Item Health Survey (version 1.0) [14]. Data were gathered through face-to-face interviews with patients, utilizing the Arabic version of the questionnaire. The trained researcher team conducted the interviews. The collected data were then entered into Google Sheets, streamlining the process for ease of handling and facilitating subsequent analysis. RAND 36 included eight health concepts, namely, physical functioning, bodily pain, role limitations due to physical health problems, role limitations due to personal or emotional problems, emotional well-being, social functioning, energy/fatigue, and general health perceptions. It also includes a single item that provides an indication of perceived change in health.

Variables

Independent variables included gender, age, marital status, children (living with family or alone), educational level, employment status, diabetes type, medication type, painful ulcer, smoking history, duration of diabetes, ulcer duration, and former ulcers or amputations.

Dependent variables included physical functioning, role limitation due to physical problems, general health perception, bodily pain, social functioning, role limitation due to emotional problems, emotional well-being, and energy/fatigue levels.

Data analysis

Analysis was done using SPSS Statistics version 23 (IBM Corp., Armonk, NY, USA). Continuous sociodemographic data are presented as mean ± standard deviation (SD), whereas categorical data are presented as counts and absolute percentages. Independent-sample t-test, chi-square and Fisher’s exact test were used to compare the findings of the study and comparative groups. Questions of RAND SF-36 were scored from 0-100, clustered, and then averaged to produce the eight subscales that reflect the health-related quality of life (HRQoL) in addition to the total score, all presented as mean ± SD. Inference was found between the groups using the independent-sample t-test to compare the QoL subscales between the two groups. Sociodemographic data were compared using Pearson and Spearman correlations and analysis of variance.

## Results

This study included a total of 120 diabetic patients (60 patients with DFU (group 2) and 60 patients without DFU (group 1)). The sociodemographic and clinical characteristics of diabetic patients with and without foot ulcers are shown in Table 1. Significant statistical differences between the two groups were observed in employment, marital status, highest academic level reached, and medicine type (p < 0.01 for all parameters). The DFU group had a higher percentage of men (60% vs. 51.7%; p = 0.389), with men predominating in both groups. The DFU group exhibited a notably higher proportion of individuals with lower educational attainment (50%), characterized by informal education and elementary school levels, compared to the diabetic group (8.4%) (p < 0.01). Among diabetic individuals with foot ulcers, there was a pronounced prevalence of insulin use (85%), indicating poorer diabetes control, whereas oral hypoglycemic agents appeared to be the popular medication within the non-DFU diabetic group (63.3%) (p < 0.01). The prevalence of type 1 diabetes was low in our sample (11.7% and 6.7% in group 1 and group 2, respectively). The mean duration of diabetes among diabetics without foot ulcers was 10.5 years and among those with foot ulcers was 11 years.

**Table 1:** Sociodemographic and clinical data of diabetic patients with and without foot ulcers in three diabetes centers in Khartoum state (n = 120). **: statistically significant. DFU: diabetic foot ulcer

Variable		DFU (n = 60) (group2)	Diabetes without foot ulcers (n = 60)	P-value
	Age (mean ± standard deviation)	52.83 ± 13.98	49.43 ± 13.99	0.208
Gender	Female	24 (40%)	29 (48.3%)	0.389
	Male	36 (60%)	31 (51.7%)	
Employed	Yes	5 (8.3%)	28 (46.7%)	<0.01**
	No	33 (55%)	32 (53.3%)	
	Therapeutic timeout	22 (36.7%)	-	
Marital status	Single	6 (10%)	11 (18.3%)	<0.01**
	Married	48 (80%)	43 (71.7%)	
	Divorced	5 (8.3%)	4 (6.7%)	
	Widowed	1 (1.7%)	2 (3.3%)	
Academic level	Khalwa education	13 (21.7%)	1 (1.7%)	<0.01**
	Elementary/Basic	17 (28.3%)	4 (6.7%)	
	Secondary/High school	12 (20%)	12 (20%)	
	College	17 (28.3%)	33 (55%)	
	Postgraduate studies	1 (1.7%)	10 (16.7%)	
Diabetes type	Type 1	7 (11.7%)	4 (6.7%)	0.370
	Type 2	53 (88.3%)	56 (93.3%)	
Diabetes duration (years)	-	11.28 ± 7.85	10.47 ± 8.96	0.596
Medicine type	Insulin	51 (85%)	22 (36.7%)	<0.01**
	Oral hypoglycemic agents	9 (15%)	38 (63.3%)	
Adherence (past 4 months)	Yes	56 (93.3%)	52 (86.7%)	0.209
	No	4 (6.7%)	8 (13.3%)	
Smoking	Yes	7 (11.7%)	6 (10%)	0.766
	No	53 (88.3%)	54 (90%)	
Ulcer duration (days)	Male	89.81 ± 135.647	-	0.188
	Female	48.92 ± 79.280	-	
Former ulcer(s)	Yes	32 (53.3%)	-	
	No	28 (46.7%)	-	
Former amputation(s)	Yes	20 (33.3%)	-	
	No	40 (66.7%)	-	

As shown in Table 2, of the eight SF-36 subscales, the DFU group scored significantly lower than the diabetic group in five subscales (physical functioning, role physical, bodily pain, social functioning, role emotional) (<0.01), yet in two subscales (general health perception, emotional well-being), the DFU scored significantly higher than the diabetic group (p = 0.041, p < 0.01, respectively). Regarding the eighth subscale, energy/fatigue, no statistically significant difference was noticed. The diabetic group had a significant overall score of 66.41 ± 18.77 versus 41.07 ± 16.02 as a total score for the DFU group (p < 0.01).

**Table 2:** Comparison of health-related quality of life in the two groups from three diabetes centers in Khartoum state (n = 120). ^a^: Data are presented as mean ± standard deviation. ^b^: Higher scores indicate less pain. SF-36: 36-Item Short Form Health Survey; DFU: diabetic foot ulcer

SF-36 subscale	DFU (n = 60)	Diabetic population (n = 60)	P-value
Physical functioning	23.50 ± 27.189^a^	74.33 ± 26.862	<0.01
Role, physical	4.58 ± 16.907	65.42 ± 38.536	<0.01
Bodily pain	61.25 ± 34.335^b^	75.50 ± 26.968	0.011
General health perception	61.25 ± 14.776	55.42 ± 16.501	0.041
Energy/fatigue	51.67 ± 14.192	50.08 ± 17.185	0.582
Social functioning	53.13 ± 32.435	78.33 ± 26.628	<0.01
Role, emotional	22.22 ± 38.162	78.89 ± 34.699	<0.01
Emotional well-being	74.20 ± 15.973	61.67 ± 17.432	<0.01
Total score	41.07 ± 16.02	66.41 ± 18.77	<0.01

As shown in Table 3, the female gender was found to correlate significantly negatively with subscales of physical functioning (p = 0.019) and general health (p = 0.011), with a total mean score of SF-36 (p = 0.038). Hence, females with DFU had worse physical functioning, general health, and total SF-36 scores than male patients. Divorced or widowed DFU patients exhibited statistically significant decreased physical pole (p = 0.037) than their married or single counterparts. The highest academic level reached was a significant positive predictor of role emotional (p = 0.04), energy/fatigue level (p = 0.021), and pain levels (p = 0.005), with higher the degree the better the results. Table 3 indicates that individuals utilizing oral hypoglycemic agents exhibited superior energy levels, emotional well-being, and total scores compared to those using insulin. The use of insulin is indicative of poorly controlled diabetes. Ulcer duration had a very strong significant positive correlation with all subscales except for the social functioning subscale. Hence, people who had developed ulcers more recently had worse scores than their counterparts who had it for a longer duration.

**Table 3:** Significant relationships between SF-36 subscales and sociodemographic parameters of the DFU group from three diabetes centers in Khartoum state (n = 60). a: Spearman’s correlation coefficient (non-parametric test). b: Pearson’s coefficient. *: Correlation is significant at the 0.05 level (two-tailed). **: Correlation is significant at the 0.01 level (two-tailed). SF-36: 36-Item Short Form Health Survey; DFU: diabetic foot ulcer

	Physical functioning	Role, physical	Role, emotional	Energy	Emotion	Social functioning	Pain	General health perception	Total
Female gender^a^	0.019^*^ -0.301^a^	-						0.011^*^ -0.325	0.038^*^ -0.269
Marital^a^	-	0.037^*^ -0.269							
Academic level^a^			0.040^*^ 0.266	0.021^*^ 0.297			0.005^**^ 0.356		
Painful ulcer^a^	0.03^*^ -0.280			0.004^**^ -0.369	0.001^**^ -0.424		-0.562 <0.005^**^	<0.005^**^ -0.460	0.001^**^ -0.426
Oral hypoglycemic agents^a^				0.004^**^ 0.365	0.032^*^ 0.227				0.042^*^ 0.263
Ulcer duration^a^	<0.005^**^ 0.541	<0.005^**^ 0.539	0.007^**^ 0.346	<0.005^**^ 0.449	0.010^*^ 0.328		.0013^*^ 0.317	0.040^*^ 0.266	<0.005^**^ 0.580
Diabetes mellitus duration^b^	0.049^*^ 0.256			0.013^*^ 0.318			0.026^*^ 0.287		0.048^*^ 0.257

## Discussion

In this study, more males (60%) than females (40%) were receiving foot care at the diabetes centers, although it was statistically insignificant. A similar finding was observed in a Malaysian study [15], which contradicted a recent study from Saudi Arabia that reported a female majority [16]. Our finding is supported by other studies from the Jabir Abu Al-Iz Diabetes Center in Sudan [9], India [7], France [17], and Norway [18]. The male predominance can be attributed to higher tobacco use and consequent vascular problems along with more exposure to vigorous and outdoor occupations, as the majority of the study’s male participants reported being manual laborers, including agricultural workers, carpenters, drivers, or merchants. According to Hjelm et al., men and women with foot ulcers may have distinct views about health and illness, which may have an impact on self-care. They discovered that men typically exhibit a more passive attitude toward self-care and preventive care, whereas women typically exhibit a more active approach [19]. Future research on gender differences in this area in Sudan is recommended.

Although statistically insignificant, the DFU group was slightly older with a longer duration of diabetes (Table 1), a common finding among similar studies [9,15,20]. Statistical insignificance can be due to the small sample enrolled. Of the DFU group, only 5% were employed during their current DFU, 36% reported taking time off of their jobs to recover, and most were self-employed. A Spanish study reported that patients are forced to leave their jobs which increases the psychological and social impact [11].

Men had considerably higher HRQoL scores than women in diabetes patients with foot ulcers, but men and women had identical HRQoL scores in diabetic patients without foot ulcers. In neither group, age had a significant effect on HRQoL. Other factors that reduced the overall SF-36 score included having a low level of education and being divorced or bereaved. Similar to our findings, in the study by Ribu et al., women reported having worse health than men [7]. Another study from Turkey showed that diabetic patients who were male, below 40 years of age, married, had fewer than eight years of schooling, resided with their families, had no complications, and had never been hospitalized had a greater QoL [21]. In another study, the average duration of diabetes in the study population was 17 ± 7 years. Notably, 70% of patients diagnosed with LLA were above 50 years of age (p < 10^-3^). Furthermore, the incidence of LLA was significantly higher (p = 0.015) in patients with a diabetic history exceeding 20 years [22].

The smallest clinically significant difference in SF-36 scores has been proposed to be a difference of 3 to 5 points [23]. The difference between groups 1 and 2 in this study was greater than 5 points for all subscales except for the energy/fatigue subscale (only 1 point), making both groups clinically similar for having low energy levels, whereas the most striking differences were for role limitation, physical (4.58 vs. 65.42 ± 38, p < 0.01), physical functioning (23.50 vs. 74.33, p < 0.01), and role limitation, emotional (22.22 vs. 78.89, p <0.01) (Table 2). Similar findings were observed in other studies [18].

The role limitation, physical scale revealed the largest mean differences between DFU patients and the comparison group. This area focuses on issues at work or with other daily activities brought on by physical health [18]. A non-weight-bearing regimen’s reduction in mobility is a significant factor affecting patients’ HRQL across various domains, including limitations in daily activities, leisure, and social contact; pressure on relationships; and feelings of stress. Additionally, DFU patients had significantly poorer social functioning scores than the general diabetes population, which may have been influenced by the patients’ reported physical limitations in everyday tasks [7].

A further significant mean difference was discovered on the physical functioning subscale of SF-36. All physical activities, including bathing and clothing, fell under this category. In previous research, the physical function scale altered the most among individuals with diabetic complications, which is consistent with the poorer physical function scores in foot ulcer patients [4,7,9,10,12,18,20,21,24].

The general health of both groups varied significantly. The effects of diabetes on overall physical functioning and health scores have been previously explored in Sudan [2,25]. More research should be done on this discrepancy, with better definitions of the diagnostic groupings and their degree of clinical severity [25]. As it goes against the findings of the previous research, the discovery that the DFU population had higher general health scores than the diabetic sample is surprising. This phenomenon can be attributed to a response bias, wherein patients may have stated their ideal state of health rather than their actual state.

The reported pain in all groups is consistent with earlier population-based research that claimed that chronic pain is a serious problem in the Sudanese population and that its prevalence is rising [2]. Despite recognizing that the findings show those with DFUs experience more pain than those who do not, there is no explanation for why this is the case. However, the same conclusions are drawn from previous studies [20]. The HRQoL of diabetic patients is severely impacted by neuropathy and unpleasant sensations, according to a recent study by Vileikyte et al. [26].

Additionally, several studies [1,2,5,10-13,27] have shown that people with ulcers report lower levels of life satisfaction than those who have diabetes but no ulcers. Our findings are in contrast to that in the emotional well-being subscale which was better in the DFU group than the other. DFU patients reported being more satisfied than their counterparts without foot ulcers, which may be due to the Sudanese social customs that reward the sick role in addition to the religious background that favors bashfulness. Although not common, a similar finding was reported in a Malaysian study [15].

Regarding the total SF-36 score in the DFU group, women scored lower than men, as women are likely to be more concerned about their health conditions and their impact on the family environment than men, particularly in families where all housework is performed by women. Such a finding was not peculiar as many studies have reported similar findings [28].

## Conclusions

In comparison to diabetic patients without foot concerns, diabetic patients with foot ulcers had lower scores in five of the eight RAND-36 subscales, higher scores in two subscales, and one subscale with an insignificant score. Similar to this, a drastically reduced HRQoL total score based on the RAND-36 was linked to diabetic feet. The results also suggest that, when compared to diabetic controls, the subscale of role limitation owing to physical issues was most affected by foot ulcers. Positive correlations were identified between ulcer duration and QoL subscales, which were noticeable.
